# A mixed methods feasibility study of nicotine-assisted smoking reduction programmes delivered by community pharmacists – The RedPharm study

**DOI:** 10.1186/s12889-017-4116-z

**Published:** 2017-02-17

**Authors:** Amanda Farley, Sarah Tearne, Taina Taskila, Rachel H. Williams, Susan Mackaskill, Jean-Francois Etter, Paul Aveyard

**Affiliations:** 1 Institute of Applied Health Sciences, University of Birmingham, Edgbaston, Birmingham, B15 2TT UK; 2 Nuffield Department of Primary Care Health Sciences, University of Oxford, Radcliffe Primary Care Building, Radcliffe Observatory Quarter, Woodstock Road, Oxford, OX2 6GG UK; 3 Department of Family Care & Mental Health, Faculty of Education & Health, University of Greenwich, London, UK; 4 Institute for Social Marketing, University of Stirling, Scotland, FK9 4LA UK; 5 Faculty of Medicine, University of Geneva, Geneva, Switzerland

## Abstract

**Background:**

Pivotal trials have established that, among people who have no immediate intention to quit smoking, nicotine replacement therapy (NRT) helps people reduce and eventually stop smoking. The prime aim of this trial was to investigate the feasibility of implementing such a programme in community pharmacies. In addition, we investigated the effectiveness of providing behavioural support compared with self-help methods and of shorter compared with standard length reduction programmes.

**Methods:**

Pharmacists were trained to deliver a smoking reduction programme and opportunistically invite people to participate in the programme. In a 2 × 2 factorial design, eligible volunteers were randomised to either receive in-person behavioural support or a self-help booklet. In both cases, participants were supported to set targets to reduce their smoking and use behavioural techniques to assist reduction. In addition, participants were randomised to cut down and stop over 4 weeks or over 16 weeks, but in either case continue NRT for up to nine months. We assessed uptake and adherence to the programme and smoking cessation four weeks and six months after a quit day and reduction in the three months following programme end and incorporated a qualitative processes assessment.

**Results:**

Only 68 of the planned 160 smokers could be recruited. Pharmacists were deterred by the bureaucracy of trial enrolment and that many smokers did not return for further support. Pharmacists sometimes subverted the randomisation or provided support to participants in the self-help arm. Smokers stayed in the programme for an average of 6 weeks rather than the 9 months envisaged. Rates of follow-up declined to around 20% of participants by 12 months. There was insufficient evidence to assess whether support or speed of reduction enhanced cessation or reduction but cessation and reduction were less common overall than in the pivotal trials for licensing NRT for this indication.

**Conclusions:**

This programme of smoking reduction and the trial design to assess its effectiveness proved unpopular to potential participants and pharmacists. As a result, the trial produced no evidence on the effectiveness of behavioural support or speed or smoking reduction. A trial of this programme in this context is unfeasible.

**Trial registration:**

ISRCTN 54805841. Registered 18 March 2010.

## Background

Historically, treatment of tobacco addiction has focused on facilitating smokers to quit abruptly. However, in the UK, in population based surveys of smokers, around 90% say they are not prepared to set a quit day, whereas around half are actively cutting down [1]. In 2012, the English Smoking Toolkit survey found that 53% of current smokers were cutting down, and 26% of reducers were using nicotine replacement therapy (NRT) to assist this [1]. In the UK, a network of specialist smoking cessation services provided in part by GP practices and pharmacies support smokers to quit abruptly [2]. Although this service has been shown to be effective [3], the rate of access is low. It is possible that diversifying the approaches available to smokers to quit may encourage more people to use specialist support. However, reduction programmes are not routinely offered as part of standard care.

There is evidence that smokers who use NRT and are supported to reduce are more likely to achieve lasting abstinence than smokers using placebo but receiving support [4, 5]. This provides proof of principle that supporting reduction enhances and does not deter abstinence in those who join such programmes. However, it leaves open the possibility that advertising smoking reduction as an option may deter people from quitting altogether. The evidence does not suggest that this is likely [6], but it remains uncertain. There is no strong evidence that behavioural support improves the rate of abstinence when offered for smoking reduction programmes [7]. Most studies showing NRT is effective have incorporated behavioural support but studies with minimal support also suggested NRT alone is effective [8, 9]. However, there is good evidence that behavioural support increases efficacy of abrupt cessation programmes by between 50–100% so it is possible that it improves success in reduction programmes [10, 11]. In this feasibility trial we examined preliminary evidence that behavioural support might be effective for smoking reduction.

The trials that led to the licence change allowing to be used for reduction asked participants to follow a nine-month reduction programme, albeit aiming to achieve a 50% reduction by 6 weeks.

However, there is also evidence to suggest that programmes of reduction over shorter periods of time may be more effective [12]. We also used this trial to examine preliminary evidence of this.

The aim of offering reduction services is to lead to more people attempting to quit and using support to do so. If even a small fraction of the 50% of smokers that are currently reducing their smoking choose to take up reduction support, the demand for services would be very great. Meeting this demand would require drawing upon the generalist workforce. Many pharmacies offer specialist cessation services already. In addition, they sell NRT, much of it for smoking reduction, and therefore they are ideally placed to offer a reduction service, but it is unclear how attractive this will be to the population and how competently these may be delivered and what proportion of people will go on to quit. The main aim of this trial therefore was to test the feasibility of pharmacists opportunistically recruiting smokers and running nicotine assisted smoking reduction programmes.

## Methods

The protocol for this study has previously been published [13]. The main aim of the study was to assess the feasibility and take-up of the programme. However, we also investigated two factors in a 2 × 2 randomised factorial trial: the speed of the reduction programme (short v standard length programme) and the value of behavioural support (behavioural support v self-help). Ethical approval for this study was granted by Birmingham East, North and Solihull Research Ethics Committee on 14^th^ June 2010 (REC reference number: 10/H1206/22).

Pharmacies were eligible to participate if they were currently running a NHS specialist smoking cessation service. Pharmacists and their teams, were trained twice for two hours to deliver the programme. Training included an introduction to why the trial was necessary, discussion of randomisation, familiarisation with the principles of reduction programmes, and also detailed the practicalities of all the stages of delivering the intervention programme and research methods from opportunistic recruitment through to the final participant visit. Pharmacists also practised their skills in recruitment and intervention delivery through participating in role plays.

### Participants

Pharmacists aimed to recruit smokers aged 18 and over who were not planning to quit smoking within the next 4 weeks but who were wanting to reduce consumption, with or without intention to stop completely. They informed participants about the study and participants signed a consent form. Participant eligibility criteria were:

1. Daily smoker with an exhaled carbon monoxide (CO) reading of at least 10 parts per million (ppm) at least 15 min after last cigarette smoked, or smoking at least 10 cigarettes or 8 g of loose tobacco as self-rolled cigarettes daily.
2. Able to be followed up by either telephone or email.
3. Not currently using pharmacological, behavioural or alternative therapies for tobacco dependence.
4. No illness that would increase the risk from concurrent use of NRT while smoking
5. Not pregnant, breast feeding or intending to get pregnant in the next 9 months
6. No severe acute or chronic medical or psychiatric condition that may increase risk associated with study participation due to concurrent smoking and NRT use or may interfere with the interpretation of study results. Most people with medical or psychiatric conditions were included.

Pharmacists were trained to identify potential participants opportunistically; they were also encouraged to use other recruitment methods such as approaching those who had tried but failed to quit smoking previously. To facilitate recruitment, pharmacies were supplied with a poster to advertise the reduction programme. In addition to the initial training, the study team also telephoned and visited the pharmacies to keep up motivation and to address barriers or concerns about approaching smokers and the treatment programmes. We also visited local general practices to raise awareness of the study and to encourage GPs to refer patients who were reluctant to quit smoking to a local participating pharmacy. GPs were given referral cards with the details of the study and details of participating pharmacies to enable this process. In the final phase of the study GPs wrote to their patients who smoked offering reduction programmes.

### Interventions

The primary aim for the reduction programme was for participants to reduce their daily cigarette consumption by at least 50%, but participants could transfer to a smoking cessation programme at any point during the reduction if they wanted to quit.

Participants were randomised to two conditions. In one condition, participants were randomised either to receive behavioural support or provided with self-help resources to guide them to achieve the same reduction. In the other condition, participants were randomised to reduce consumption rapidly, halving consumption by two weeks, compared with the standard reduction schedule, halving consumption by six weeks.

#### Behavioural support vs self-help

In the behavioural support arm, pharmacists explained the rationale for the programme by suggesting that learning a new pattern of smoking would prevent consumption increasing again by disrupting learnt associations between cues and smoking behaviour. They encouraged participants to use NRT and choose one of three methods of reduction, called the “timer method”, “smoke-free periods”, or “unstructured”. In the timer method, participants used a timer, such as a mobile phone, to signal when they could smoke and agreed to smoke only when the timer indicated it was appropriate to do so. This time lengthened on each occasion a person wanted to reduce. The smoke-free periods method divided the day into hours and participants progressively eliminated hours, agreeing not to smoke in the hours participants designated smoke-free. In the unstructured method participants were free to smoke when they liked but to set aside each day’s cigarette ration in a pack. We encouraged pharmacists to use either of the structured methods in preference because there is evidence that they are more effective [14].

In the behavioural support arm, participants returned to the pharmacy for eight occasions after baseline for support. We envisaged pharmacists spending around 10 min on support on each occasion.

In the self-help arm, the smoking reduction methods were exactly the same as above, but they were explained in a written booklet. We asked pharmacists to hand out the booklets without further advice or interaction.

In the behavioural support arm, pharmacists enquired about participant’s willingness to quit smoking at each visit and transferred a person ready to do so to a standard smoking cessation programme. In the self-help condition, the booklet prompted the reader to consider this.

#### Short vs standard length reduction programmes

Both programmes contained 8 steps, whether offered in conjunction with a self-help booklet or with support from the pharmacist. Initially, we asked participants to try to reduce consumption by a quarter, then by a half, then three quarters to achieve abstinence by the fourth step. In the short programme, this was scheduled to take four weeks, in the standard programme 16 weeks. The pattern of visits in the standard condition was scheduled as baseline, 2, 6, 10, 16, 22, 28 and 34 weeks, and in the short condition as baseline, 1, 2, 3, 4, 6, 8 and 16 weeks, with the first four visits marking a step with a new reduction goal if the last had been achieved. If a participant did not want to achieve abstinence at the designated point in the programme (i.e. at 4 or 16 weeks) then the additional visits were to motivate further attempts at reduction and/or cessation.

#### Adjuvant pharmacotherapy

In all treatment conditions, participants were prescribed NRT throughout the programme. Pharmacists encouraged participants to use both a nicotine patch and a short acting form of NRT (2 mg gum, 2 mg sublingual tablets, 2 mg lozenge, inhalator or nasal spray) and to continue using NRT regardless of their success in reduction, unless they had moved onto a cessation pathway and had successfully quit. The protocol specified that participants smoking less than 10 cigarettes per day (cpd) should be prescribed 7 mg/24 h or 5 mg/16 h patches, 10–19 cpd 14 mg/24 h or 10 mg/16 h patches and 20+ cpd were prescribed 21 mg/24 h or 15 mg/16 h patches. Participants were advised to replace each ‘missing’ cigarette with use of one dose of their chosen short acting product. The rationale for this regimen is described in the trial protocol [13]. All participants were encouraged to use NRT for 9 months regardless of intention to reduce or stop, or failure of either reduction or cessation.

### Randomisation sequence generation, allocation concealment, implementation

The research team generated the randomisation sequence using a computer algorithm at http://www.randomization.com, and the allocations were given to pharmacists in numbered, sealed envelopes. Block randomisation stratified by pharmacy was used to generate the sequence, with two randomly ordered blocks of four and one of eight. This ensured balance of participants within a pharmacy. Participants were randomised with a ratio of 1:1 for both of the two conditions.

### Follow up and blinding

Data on reduction or cessation were collected by pharmacists when participants visited the pharmacy for study visits. Regardless of whether participants visited the pharmacy we assessed cigarette consumption, NRT use, and cessation each month by email containing a link to an online questionnaire. We elected to use an online questionnaire to reduce the sense that the participant was being monitored by a caring person which could have inadvertently provided behavioural support. Two reminders were sent to participants that did not complete the questionnaire, after which participants were telephoned. Participants who claimed abstinence for either 4 weeks or 6 months were asked to attend the pharmacy for carbon monoxide verification and were compensated £20 for doing so.

It was not possible to blind pharmacists or participants to the treatment allocation. However, the telephonists conducting the monthly follow up calls and hence the person assessing the outcome assessor was blind to treatment arm.

### Qualitative interview data collection

Semi-structured interviews were conducted with 10 pharmacists at the end of the study to investigate key barriers and facilitators to opportunistic recruitment and implementing the reduction programme, and their overall response to the programme and trial. Pharmacists chosen for interview reflected both those who had recruited many and few participants. Semi-structured interviews were also conducted with trial participants, with purposive sampling from each trial arm of participants who dropped out, reduced or quit smoking. These interviews covered participants’ motivation for agreeing to participate, participants’ views on how participating in the programme had affected their smoking, why they felt their smoking had been affected, and also their experience of participating in the trial, including the monthly follow up. Interviews were audio-recorded, transcribed and anonymised before analysis.

### Outcomes

The main outcomes assessed the feasibility of implementing the reduction programme in pharmacies, and estimated important parameters necessary for designing a definitive trial. These included: (1) the percentage of pharmacists approached that agreed to participate and percentage of participating pharmacies that passed an assessment of competence; (2) monthly recruitment rates of smokers onto the reduction programme; (3) proportion of smokers who moved onto a cessation pathway or who dropped out; (4) quantitative description of fidelity of the pharmacist to the treatment conditions; (5) the number of people that completed the monthly questionnaire follow up, and the number contacted by telephone; (6) the amount of NRT used by trial arm; (7) number of adverse events and (8) the proportion of participants that would recommend the smoking reduction programme to another smoker.

Qualitative process outcomes included: (1) qualitative description of fidelity to the protocol of pharmacist-delivered behavioural support sessions; (2) pharmacists’ views regarding the design and running of the programme; (3) participants’ (smokers’) views regarding the design and running of the programme.

In addition, we collected efficacy outcomes defined as biochemically confirmed “floating” sustained abstinence for four weeks. Sustained abstinence was defined using the Russell standard definition of prolonged abstinence i.e. self-report abstinence or up to five cigarettes smoked only, from day 15 after quit day onwards [15]. The beginning of the period of sustained abstinence could be at any time (i.e. floating) during the time a participant was in the study [16]. Self-report abstinence was biochemically validated if the participant’s exhaled breath had a CO concentration of less than 10 ppm.

Secondary efficacy outcomes were biochemically confirmed prolonged abstinence measured at six months, self-reported abstinence at four weeks or six months, and sustained smoking reduction. Smoking reduction was measured in the three ways: (1) self-report reduction in daily cigarette consumption to <50% of baseline value sustained between months 9–12 after baseline; (2) self-report daily cigarette consumption lower than baseline consumption by any amount which was sustained between 9–12 months after baseline; (3) mean difference between baseline and 12-month daily cigarette consumption. For (1) and (2), if one middle datum point was missing, but all others showed <50% reduction, this was counted as a sustained reduction. If the end data point or more than one data point was missing, this was not counted as sustained reduction. For (3), if 12 month follow up data was missing, this was imputed as baseline daily consumption. A sensitivity analysis was also performed using last observation carried forward.

#### Sample size

Given that the objective of this study was to test feasibility, the trial was not powered to provide definitive evidence of the effectiveness of one reduction programme versus another. The most important outcome in terms of health is proportion of participants who move on from the reduction programme to quit. We calculated that 160 smokers (16 patients per pharmacy) would provide sufficient power to detect plausible differences between arms in abstinence rates at 4 weeks. Our previously published systematic review showed that in a standard length reduction programme, 7% of participants sustain abstinence at 6 months [4]. This would equate to about 21% achieving 4 weeks abstinence. A previous trial suggested that shorter reduction programmes are about twice as effective as standard length programmes [12]. Based on these estimates, a sample size of 160 would have 80% power to detect a risk ratio of 1.7 or 90% power to detect a risk ratio of 1.8, which is lower than that observed in the Haustein trial [12]. We also considered that this sample size would be sufficient to explore the additional process outcomes. For example, if attendance for behavioural support session was 63% (as observed in our review [4], with 80 smokers receiving behavioural support, we could estimate this with +/−11% precision with 95% confidence.

#### Statistical methods

Quantitative process measures were analysed using standard descriptive statistics, and compared by trial arm where appropriate. Relative risks and risk differences with 95% confidence intervals were calculated to compare efficacy outcomes (i.e. sustained cessation or reduction), between trial arms. As we stratified the randomisation by pharmacy, we accounted for this in a generalised linear mixed model, with pharmacy set as a random effect to allow generalisation and treatment arms as fixed effects using a logit link function and robust covariance matrix. These analyses were conducted as an intention-to-treat analyses, where participants with missing data were assumed to be smoking or to have not reduced, and all participants randomised to a condition were included in the denominator. To compare continuous outcomes between trial arms (i.e. mean daily cigarette consumption), we calculated the difference in means and compared these using a *t*-test. We planned to conduct multi-level modelling examining whether the availability of a reduction programme increased or decreased the number of people attempting to stop smoking in the pharmacies; however, we were unable to obtain the necessary data from the local stop smoking services.

Qualitative data generated from the semi-structured interviews were analysed using thematic analysis. Themes were derived deductively from the aims of the interview, and also arose from the interview data.

## Results

**Fig. 1:**
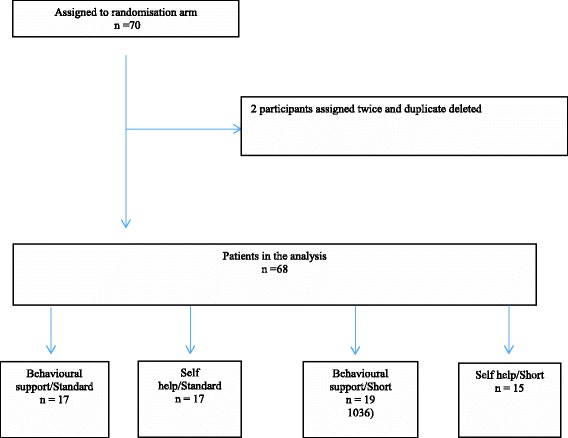
Study flow

The original intention was for 10 pharmacies to each recruit 16 people in four months, which all agreed was a feasible target. In the event, participant recruitment fell way short of this so we recruited another batch of pharmacies. Altogether, we approached 27 pharmacies of which 18 (66%) agreed to participate. Pharmacists all attended both training sessions and demonstrated sufficient competency during role playing to be considered successfully trained. There were a total of 68 participants recruited into the study between Dec 2010 and Sept 2012 (Fig. 1). Several initiatives were instigated throughout recruitment to boost recruitment; however, this final figure did not meet the recruitment target.

### Quantitative process measures

#### Monthly recruitment rates and randomisation

**Fig. 2:**
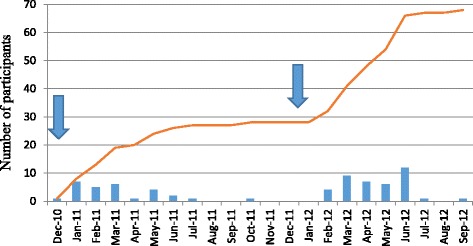
Monthly (bar) and cumulative (line) recruitment rates over the study period. Arrows indicate timing of recruitment drives

A median of one (range 0–12) participants were recruited each month (see Fig. 2). GP mailings to smokers to encourage them to join the programme recruited few additional participants.

#### Baseline characteristics

Low recruitment meant that the block randomisation did not produce perfect balance and there were imbalances in the characteristics of participants across study conditions at baseline, particularly for gender, ethnicity, number of cigarettes smoked per day, and the participants’ reasons for wanting to cut down (Table 1).

**Table 1:** Participant baseline demographic and smoking characteristics

	Behavioural/Standard *n* = 17	Self Help/Standard *n* = 17	Behavioural/Short *n* = 19	Self Help/Short *n* = 15
Age yrs, mean (SD)	44 (12.3)	44 (16.5)	44 (12.0)	43 (13.1)
Male, n (%)	11 (65)	7 (41)	6 (32)	10 (67)
Ethnicity, n (%)				
White	7 (35)	8 (47)	11 (58)	6 (40)
Other	9	7	5	7
Missing	0	0	0	1
Age started regular smoking, mean (SD), n	16 (3.0), 16	17 (5.0), 14	16 (2.3), 16	17 (2.6), 14
Have you ever made a serious quit attempt, n (%)				
Yes	12 (71)	12 (71)	12 (63)	11 (73)
No	4 (24)	2 (12)	4 (21)	3 (20)
Missing	1 (5)	3 (17)	3 (16)	1 (7)
FTND, mean (SD)	5 (2.3), 16	6 (1.7), 15	6 (2.8), 16	6 (2.5), 14
Cigarettes per day, mean (SD)	17 (8.2)	23 (8.2)	20 (9.1)	17 (6.5)
CO, mean (SD), n	17 (8.4), 17	19 (11.2), 17	19 (10.4), 18	17 (8.7), 15
Reasons for cutting down, n (%)				
I want to cut down as a way to stop	9 (53)	8 (47)	10 (42)	11 (73)
I want to cut down as a way to smoke less	7 (42)	7 (41)	6 (32)	3 (20)
I’m not sure why I want to cut down	0 (0)	0 (0)	0 (0)	0 (0)
Missing	1 (5)	2 (12)	3 (16)	1 (7)
Highest educational qualification				
Secondary school (up to age 15/16)	7 (41)	4 (24)	7 (37)	6 (40)
Sixth form (up to age 18/19)	4 (24)	2 (12)	1 (5)	1 (7)
Professional or technical qualification/diploma after school	0 (0)	1 (6)	1 (5)	0 (0)
University/polytechnic degree course	0 (0)	2 (12)	0 (0)	1 (7)
Still in full time education	1 (6)	2 (12)	2 (11)	3 (20)
None of the above	4 (24)	4 (24)	5 (26)	3 (20)
Missing	1 (6)	2 (12)	3 (16)	1 (7)

#### Fidelity to randomisation

There was evidence that pharmacists did not follow randomisation protocols. We discussed the necessity for randomisation at the training, although it was clear that pharmacists believed strongly that their support was essential. In both cases of duplicate enrolment, pharmacists opened a second envelope in order to offer behavioural support.

#### Fidelity of the pharmacist to the treatment conditions

Participants allocated to behavioural support were randomised to receive sessions over a standard length schedule (0, 2, 6, 10, 16, 22, 28 or 34 weeks) or a short length schedule (0, 1, 2, 3, 4, 6, 8, 16 weeks). The median number of weeks after baseline (Visit 1) that participants returned for behavioural support was similar to these schedules specified in the protocol. However, the range was large, and for some participants the behavioural support plan was not adhered to (Table 2).

**Table 2:** Fidelity to standard and short behavioural support schedules

	Behavioural Support/Standard *n* = 17			Behavioural Support/Short *n* = 19		
	n attending	Week after baseline as specified by protocol	Actual attendance time (weeks) median (range)	n attending	Week after baseline as specified by protocol	Actual attendance time (weeks) median (range)
Visit 1	17	0	0	19	0	0
Visit 2	11	2	2 (1, 6)	15	1	1.3 (1, 9)
Visit 3	9	6	5.4 (2, 9)	12	2	2.1 (2, 17)
Visit 4	8	10	9.4 (3, 48)	10	3	3.4 (3, 7)
Visit 5	7	16	14 (4, 52)	7	4	5 (4, 8)
Visit 6	6	22	21.7 (5, 59)	5	6	7 (6, 11)
Visit 7	4	28	27.4 (6, 64)	5	8	8.7 (7, 18)
Visit 8	2	34	7.5 (7, 8)	4	16	17.5 (11, 27)

Pharmacists were asked to set a reduction target with participants in the support arm to achieve by the next meeting and did so in a median of 92%, (range 33, 100%) of participants who attended Visits 1–8). Pharmacists were asked to discuss a reduction method with participants and record this and did so in a median 89% (range 45, 100%) of participants who attended Visits 1–8 (Table 3).

**Table 3:** Number of participants in behavioural and self-help arms who had a target and method recorded.

	Behavioural *n* = 36				Self Help^a^ *n* = 32			
Visit	n	Last visit target met *n* (%)	New target set n (%)	Method recorded *n* (%)	n	Last visit target met *n* (%)	New target set n (%)	Method recorded *n* (%)
1	36		31 (86)	36 (100)	32		9 (28)	23 (72)
2	26	26 (100)	26 (100)	24 (92)	17	8 (47)	10 (59)	10 (59)
3	21	21 (100)	18 (86)	19 (90)	14	7 (50)	2 (14)	6 (43)
4	28	18 (100)	17 (94)	16 (89)	9	4 (44)	2 (22)	4 (44)
5	14	14 (100)	13 (93)	13 (93)	7	5 (71)	1 (14)	2 (29)
6	11	11 (100)	11 (100)	5 (45)	5	4 (80)	2 (40)	2 (40)
7	9	9 (100)	8 (89)	7 (78)	4	4 (100)	0 (0)	1 (25)
8	6	6 (100)	2 (33)	4 (67)	3	3 (100)	0 (0)	0 (0)

^a^Participants randomised to self help should not have undergone a review of their previous targets, setting of a new target or discussed method of smoking reduction with the pharmacist. However, there was evidence that this was done in a large proportion of these participants

#### Contamination in the self-help arm

Participants randomised to the self-help conditions should not have received behavioural support when returning to collect further NRT prescriptions. However, there was evidence that pharmacists were routinely recording reduction targets, setting new reduction targets, and less commonly recording reduction methods used in the self-help participants, suggesting that they were in fact providing support (Table 3).

#### NRT use

The protocol advised use of NRT for up to 9 months as long as participants were continuing to try to reduce. However, participants were prescribed NRT for a much shorter time, which was similar in all arms (median (range) months: short 1 (0.25–13), standard 1.75 (0.25, 15)), behavioural 1.25 (0.25–15), self-help 1 (0.25–14).

Six participants were prescribed NRT for longer than 9 months (up to 15 months).

Fifty-eight of the 68 (85%) included participants were prescribed patches. Participants in each arm were prescribed a similar number of patches per month, and the median number prescribed was similar to the recommended amount (median (range) patches/month: short 28 (3–28), standard 26 (2–29), behavioural 28 (2–29), self-help 26 (7–29)). Forty four participants using patches were also prescribed a median (range) of 1 (1–3) additional short-acting NRT product. Participants used inhalators, lozenges, gum, microtabs, mints, nasal spray and minis, and the most popular oral product was the inhalator.

#### Follow-up

**Fig. 3:**
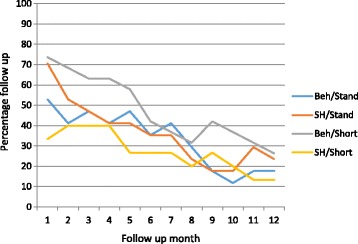
Monthly percentage follow up rates by trial arm

Most participants did not complete the online questionnaire and instead monthly follow up was conducted by telephone. The percentage of participants successfully contacted each month fell steadily in all trial arms, with 18, 24, 26 and 13% being followed up at 12 months in the behavioural/standard, self-help/standard, behavioural/short and self-help/short groups respectively (see Fig. 3).

### Qualitative process measures - pharmacists

Ten pharmacists were interviewed at the end of the trial about their views about the programme, and barriers/facilitators to recruitment to the trial and participation in the reduction programme. There were no clear differences in the themes emerging from pharmacists who had been able to recruit patients and those who had not.

#### Views about the programme (Table 4)

**Table 4:** Attitudes towards the reduction programme – illustrative quotes from pharmacists

*“I think people don’t come at the moment because they think that they’re going to be pressurised… you know and a lot of finger pointing…so I think it is a good idea to have a programme that… allows very heavy smokers…to reduce first, before they quit, and that might help them more. ” PH01*
*“I think there is definitely a place for it…it would be able to help a lot more people than I was presently because the stop smoking services is very strict really… whereas at least with [the reduction programme] they can approach it gradually” PH06*
*“I think it’s quite important to establish that when you set out with a reduction scheme, you’re on…what I think is dodgy grounds because the patients thinks… “well I can reduce…and they don’t, they don’t see that final goal as quickly, whilst the cessation scheme is more likely… I think just as long as…it’s set out from the beginning that you’re going to reduce to quit, then I think…it can be valuable part of the NHS.“ PH15*
*“With the Self Help… I think it just depends on the person…how much enthusiasm they’ve got for it…if they’ve got… to do it themselves and make their own decisions, I don’t think that motivated them at all“ PH04*
*“I think it’s the smoke free period without a doubt [that is the best approach]. The timer method is only suitable for, very small proportion of the population. And even then, it doesn’t actually deter people, it might actually work the opposite way, and people may be used to then smoking every hour. Or every hour and a half, they make it the habit, that, ‘Oh God… I need to smoke now.” PH07*
*“When you are helping the person, behavioural support, it was more satisfying than…just tell them to go… do it themselves…”PH10*
*“I think, the one to be left on their own to do everything would have been…not appropriate, I think they really needed the support.”PH12*
*“It was a very thin line between ‘I am giving you support now,’ where ‘I’m not giving you support now.’ Now where do you stop, giving that support…You can even sit down with the guy and say, yeah try here, try here, do this, like we do with NRT product. But you can’t give him support…it’s a very thin line between, now I’m giving him support… and kind of messing with the study, where does my role stop, where can I say, nope, I can’t give you any more support past this point.” PH16*

All pharmacists felt that the reduction programme was good in principle, and that participating in the study would be a good opportunity to extend the smoking cessation services already successfully offered by the pharmacy. Pharmacists felt that the programme was useful because it would offer a less strict option to cessation and would suit patients who had not been able to quit using the cessation service or for heavy smokers who may not feel able or ready to quit. In this way, they had anticipated that by participating in the programme, they would be able to reach more of their patients who found it difficult to quit smoking abruptly. Only one pharmacist expressed concern that a reduction programme may divert smokers away from quitting, and he believed that it was important to be clear to participants at the start of a reduction programme that the purpose was to cut down in order to quit.

Most pharmacists reported that they preferred offering behavioural support rather than the self-help programme. Behavioural support was seen to be more satisfying, pharmacists believed it would be more effective and did not feel comfortable assigning patients to self-help. Generally, pharmacists had no strong views on the behavioural methods to help people reduce. However, some felt that the timer method was not feasible, with one pharmacists suggesting that it might be counter-productive to encourage people to smoke when it was the correct time to do so. Some felt that smoke free periods or unstructured methods were more popular and practicable.

#### Recruitment methods (Table 5)

**Table 5:** Recruitment methods - illustrative quotes from pharmacists

*“People that came in and inquired about smoking patches and… inquired about what they could do for smoking, I would offer them [the reduction] programme alongside the [cessation] programme” PH01*
*“We’ve had the posters around the window, we’ve tried moving it about to different locations thinking it would maybe catch their eye better at different locations, we’ve also when people come into enquire about stop smoking we do ask them would you like to cut down or are you looking to just… quit altogether” PH2*
*“I know there was one GP he would send these letters out…we only got like four people ring us out of the whole lot. And only…one of them turned up out of the four. So, it just didn’t get people on as much as you’d hoped.” PH04*
*“We put up a poster in all the waiting rooms in surgeries, we put posters up in pharmacy, and people who were coming in, we were asking them if they wanted to give up smoking or did they want to reduce smoking. So, those are the main [recruitment] methods we used.” PH06*
*“We get a lot of referral from our GP, as well as…approaching patients… I gave it as a dual option, they could go for? the reduction method or they could go for the straight NRT approach.” PH08*
*“the record I keep of the, over the years of stop smoking service, I rang them up and I said, were they still smoking and, would they be interested, and also when we had some customer who came in and we asked them would you like to join this programme, when they bought a prescription and we’d normally ask them if they smoke, or even if they buy a cough mixture ….we targeted about fifteen people.. all of them, declined…we gave out lots of cards… I dropped it in the local surgeries as well but we didn’t get anything back from the surgeries. No referrals or nothing.” PH10*
*“we tried as much as possible to promote it, the posters and the little cards inside the things and stuff… it was mainly just anybody who…came to us to say that they noticed the poster … I say ‘look we offer two schemes… there’s one that you can…just … cut down, or there’s one that you can stop altogether, what would you prefer” PH15*

Pharmacists main recruitment approach was to target people that they had tried to help to stop smoking abruptly and who had failed. They also described offering the programme to patients who asked for help to stop smoking, bought nicotine replacement therapy or had seen poster advertisements in the window of the pharmacy or at their local general practice and enquired about the programme. Although pharmacists and their teams were trained to broach the subject of smoking reduction opportunistically with customers whom they knew smoked, only one pharmacist described using this technique.

#### Barriers to recruitment and continued engagement with the programme (Table 6)

**Table 6:** Barriers to recruitment and continued engagement – illustrative quotes from pharmacists

*“the initial consultation takes a bit of time… and because people haven’t been on this programme before, it’s a lot of information to go through whereas the other form [for cessation], a lot of people come in and re-sign up… it’s been running for years so they know all about it, and this is new so, they’ve just a bit more time to get their head around it and explain everything to them and get my head around it as well…What I don’t like about it at the moment is the fact that the whole paperwork and the structure for it is different to the other service…[if] the paperwork was similar, it would be a lot easier… this is all quite new and then… you’re slapping around trying to get the different paperwork and sometimes I think you… just leave it to the patients to come back…if I could… get an appointment from them there and then and get a telephone number…I think that maybe would’ve been a better thing to do. ”PH01*
*“I think when you try and explain the structured method to them…smoking every so many minutes…I think they look at you a little bit funny… I think they expect to go on the programme and just use the programme to reduce the number of cigarettes themselves and then when you sit and explain all the structure to them it sort of baffled them a little bit” PH01*
*“I think because of the staffing issues as you’ve seen and the fact of the length of the interviews as well for the first few appointments that’s probably a put off… but with a lot of our patients they’ve not really actively come in and seek to cut down, I think when they come in they’ve made up their minds they want to quit, they want to go all the way… so I think that’s probably the biggest hurdle we’ve had.” PH02*
*“It was hard to get people on. … they know there is a stop smoking course, and they will come because they want to stop smoking today, but I don’t think a lot of people wake up saying, “I want to cut down…I did ask people, even the people that came in… “Oh, I want to stop smoking” I’d tell them that we were doing the reduction or to stop smoking and they say, “I want to stop smoking, not the reduction.” PH04*
*“I mean, the cessation programme is short and you have to stop by the second week; whereas this, you get people who go up and down, up and down over the longer period, you know…I think if they had like a barrier, say, ‘By this time you have to have stopped or cut down to this much’, then, I think they will try a bit harder…they knew that they can go up and down and you know it wouldn’t really matter, I think that’s when that probably took advantage of.” PH04*
*“During the period that it was running we actually managed to recruit about ten people into the stop smoking service, but when I offered… those same people the choice of joining the Redpharm study, they all declined and the reason they declined was because when they were given the choice about being supported or not supported, they didn’t want to take the risk, of being not supported.” PH06*
*“the reason [that we had difficulty recruiting] I think is it’s a very highly ethnic area and English language isn’t number one medium so with the paper work and the whole bulk that came with it, it was very difficult to explain it to them how the programme worked. So we had initial interest but then nobody carried on… when I explained it further they weren’t really interested because of the technicalities of it…English isn’t their number one language…to explain something that is fairly complex to them it took my time and takes their time …I know trials you have all these technicalities that have to be followed but if it was the programme though we could just go straight into it without the issues surrounding it I think it would work definitely… I did try and recruit, well the initial recruitment as in speaking to them and I also offered them our PCT programme [cessation], which is the standard 12 week programme and they’d rather go for that one.” PH08*
*“Once you get about eight, nine people on it, it’s… too… time consuming, if it’s..a simple thing just filling the chart, sign it, away you go, it would’ve been easier for me. But… to keep you in behavioural support or, getting people in, saying look how’s it going,…it was time consuming….it just meant that I had to cap the number of people I could take on” PH07*
*“people didn’t have the understanding that it’s not offered everywhere, a lot of them thought, oh, we’ll go down road and we’ll do it there, or we’ll go closer to home and do it there… and a lot of them… whenever you hear research they think, oh we’re guinea pigs, what’s going happen are you going to give us special drugs, but apart from that, which was a small portion, there wasn’t any problems” PH0*
*“If you give people the choice between stopping smoking and reduction, they want to stop.” PH10*
*“The, paperwork is a problem to pharmacists. It’s a bit long and it takes a little long to fill…we’d rather to do it immediately you may try to give someone an appointment to come the next day, in doing so that person may not come so you may end up losing that, and that’s where I find a little bit of a problem. We’d like a shorter level--I know you’re going to collect statistics but, we have to think of a better way to be, to have less paperwork…I think the level of education of most people… possibly was not sufficient for them to understand in detail what this programme was doing to them…There is a big document…in the beginning, and there were, for the patient, the bigger documents that they signed in the beginning, I think some of them don’t like to sign documents, that’s another thing” PH10*
*“I mean, once they starting losing interest that was it, you know, even the behavioural support people, they would…once they got past the initial appointments, they were just coming in every so and so just to get patches and it’s just one of those things. I think they felt that they weren’t doing anything like on the programme or something…They started off well and after three to four weeks…it just went back up…they would come in and say, “Oh, we’re going to try and do this.” …but then they just go back to normal and then they wouldn’t turn up. So, they start at a level, they go down for a few weeks and then they go back up and then just disappear.“ PH04*
*“a lot of people left half way because maybe they didn’t see the point, or they didn’t understand the forms that they had to fill, and I can understand but most of them they’d not finished completely so only for four weeks, after that they were tired and they stopped. I have time for them when they come to the pharmacy, but I haven’t got time to start making phone calls” PH10*
*“when we mentioned to them it’s a year programme… they were a bit reluctant, initially when we say to them, look this is a good programme, you don’t have to quit…straight away, it’s a gradual process so they were impressed it, and then when I gave the information of the little bits, the first, will be about forty five minutes and then it’s for a year you have to really be committed to this programme, then they become reluctant, but the four people I talked to, who’d really benefit from it, they just declined on this, principle… if you were to roll this programme out you probably might need to reduce the number of questions that have been asked, and the… timeframe of the first session, probably needs … at least, fifteen minutes reduction. I think you can get comfortably half an hour somebody come to sit with you, but I think… then, it’s become like a barrier for them, for us to get them to sit twenty minutes in the first session when we do other stop smoking services, it’s already a task….so…”PH12*
*“after talking to the clients, all gave me that reason is that, this first interview’s too long for us and, we can’t give that much time. And, the other problem is, a lot of the clients here, their English is not, fluent English… we have to speak in their own languages, either in Bengali or, Urdu like that so that’s become, little bit hard.”PH12*
*“…because we’re offering a smoking cessation scheme as well …I’d give them the option… about which one they wanted to go for, and they all seemed to go for the cessation scheme rather than the reduction scheme, so … didn’t really seem to work over here very well.” PH15*
*“The cessation scheme is quite straightforward, there’s a bit of paper, the patient comes in, you take a quick… CO reading, you discuss the plan with them, and they just literally come in week in week out to collect their NRT, keep an eye on them… I never really got to have a good, chance with the reduction scheme, but it looked complicated from the outset, that’s my initial view… it’s a randomised trial isn’t it…so obviously, it can’t be as straight forward as a cessation scheme but because of its nature I guess, it just seemed extremely complicated from the outset.” PH15*
*“they were..like, oh we’re here to stop, but we want a nine month course, and we want your support. And it’s like, well we can’t always, it depends, if you get that, in a raffle, kind of way. And a lot of people say, oh well we’ll go on another one then ’cause, eight to ten weeks we can do that, so we had a lot of problems with that especially around this area, with people didn’t understand that.” PH16*

Pharmacists described several barriers to successful recruitment and engagement with the programme.

#### Desire to reduce

Most pharmacists reported that when they offered patients a choice between joining the smoking cessation programme or the reduction programme, patients chose cessation rather than reduction.

#### Length and complexity of initial consultation

Pharmacists all felt that the length of the initial appointment and the complexity of the paperwork were major barriers to recruitment. This, in the pharmacists view, had put patients off becoming involved in the programme, and some pharmacists also indicated that it had deterred them from recruiting. In particular, they felt time was wasted if participants didn’t return for subsequent appointments. Although pharmacists acknowledged that the length and the complexity of the first appointment was necessary to administer the research requirements of the programme (i.e. assess eligibility, ensure informed consent, and randomisation), they compared the requirements to administering the smoking cessation programme which was considered to be more straightforward.

#### Understanding of research

Some pharmacists also felt that patients didn’t understand, or were not prepared to accept, random allocation to a treatment condition. In particular, pharmacists felt that patients did not want to “take the risk” of being assigned to self-help. Some pharmacists felt concerned that patients would feel suspicious of the research process, feeling that they were being experimented on and that this might put them off wanting to take part. Some pharmacists also suspected that patients did not understand that this programme was not widely available and that this was an experiment. As a result, they felt that people did not understand the imperative to enrol straightaway.

#### Length, complexity and administration of the programme

Pharmacists felt that an important reason that patients chose the cessation programme over the reduction programme was because the former was simpler, shorter and more familiar to patients. Pharmacists felt that patients didn’t want to reduce over months, but were motivated to make more immediate changes. Some pharmacists decided not to recruit their quota of 16 because they did not have the staff time available. The length of the programme, and also the complexity and amount of paperwork involved were also perceived to be barriers in maintaining enthusiasm for the programme. Pharmacists reported that some patients were deterred by having to sign consent forms to participate in the trial and they found it difficult to comprehend the whole programme. This was particularly highlighted to be a barrier for patients whose first language was not English and those of lower educational level. Pharmacists acknowledged that the administration involved was necessary to fulfil research requirements, but felt that if such a programme was to be implemented it should be administered in a similar way to the cessation programme.

### Qualitative process measures - participants (Table 7)

**Table 7:** Main themes and illustrative quotes arising from interviews with participants

Recruitment
“[The pharmacist] knew that I smoke anyway. So, he just suggested it to me as an idea, because I always buy patches from the pharmacy. So, he goes “Why not do it this way, because it would save you money and it would help you as well.” PPT1
“I went in and asked him because he got the thing on the door… about quitting there and to ask for advice there. And, I asked him. And, he says, “I’ll put your name down.”…And, that was it. And, it just sort of picked up from there.” PPT2
"Normally, what it is with these local chemists, they always have programmes running. And, I’ve been with him once before or twice before, I think, previously. But, he knows that I give in to temptation very quickly – the smoking part of it…I felt happy [that he offered me the programme], because …it’s actually not just like a face behind the counter…just give the medication and that’s it. He actually showed concern.” PPT3
“I [saw] the sign outside, I was sort of interested myself anyway” PPT4
“I was asked because I’ve been buying patches and stuff previously. So they obviously knew that I was trying to give up.” PPT5
Reactions to the programme
“It was overall a good experience being on it because …I was buying patches before privately and they were costing too much. So, obviously when I found out about this scheme, it’s a one-off payment and then you’ve got like nine months as a free course thing so I took part of it and it did actually help me progress quite well actually. I’d been on 8 weekly programmes before, you see, and they haven't been successful. Whereas, this one …it’s got more time for me to quit…or to reduce, either way” PPT1
“***Do you think that the programme worked for you overall?*** Yeah, it did. ***…and why was that?*** Well, you know, it just did. We just made our minds up well I did. And, I just stopped.”PPT2
“**Do you think that the programme worked for you?** It has. It actually made a lot of great improvement for myself. Again, I’ve cut down. Sadly, I haven’t completely quit but, I have actually cut down to between 2–3 a day; now, to maybe once every 2 days……and that’s without any patches or anything. So that’s actually… worked for me. It’s like I have a bit more…like a stronger hold on it.” PPT3
***“Did you find it helpful that you didn’t have to stop completely?*** I did stop completely. The thing was I did stop completely, I didn’t sort of go smoke and…because, you were allowed to have a certain amount of cigarettes a week. But, from Day 1, I sort of went cold turkey and used the patches. And, I didn’t smoke.“PPT4
Motivation
***“Did the offer to join the programme…increase or affect your motivation in any way to try and quit or reduce your smoking?*** Increased it, because I knew that it was there in place and I could take part in it. I had an opportunity so I worked for it… PPT1
“You’ve got some support, haven’t you? If you tried to do it on your own, you’ve got no support. Whereas, if you’re doing it like with… any pharmacy… you start to get educated. You could go in and have a chat and you’d get it all sorted…. It gives you, you know, another incentive to keep going.”PPT2
“It’s monitored on a weekly basis. Yeah, that was an incentive really. It’s monitored. Normally, the other ones don’t really get monitored. It gives you a greater incentive to not to smoke. And, especially, when you see the carbon coming down and down every week or when it hits zero” PPT4
***“When you first joined the programme, did it help your motivation?*** They did a bit, yeah, of course, you’d have to go through all the process of filling in forms… and talking about it and they ask you all the necessary questions … “What have you tried and have you tried this or have you tried that?” And all the details about how you manage…with those things that you tried and I thought, “Oh, yeah, I will give it another go.” But it didn’t last long, I’m afraid, now” PPT5

Five participants were interviewed about their experience of taking part in the programme (Table 8). All participants were motivated to quit when they joined the programme, and had either approached the pharmacists because they wanted to stop smoking or the pharmacist had approached them because of a history of struggling to quit or they were buying patches from the pharmacy. The offer of taking part in the programme increased participants’ motivation to try and quit, as it presented them with an opportunity to do so. One participant also noted that they had found answering the questions on the accompanying forms motivating.

**Table 8:** Participants characteristics

ID	Treatment	Gender	Smoking status	How recruited
PPT1	Standard/ Self-help	F	Quit on the programme but had relapsed by time of interview	Known smoker to pharmacist – pharmacist asked if she wanted to join the programme
PPT2	Standard/ Behavioural	F	Quit on the programme and still abstinent at interview	Saw the poster advertising programme to quit and asked to be enrolled
PPT3	Standard/ Self-help	M	Reduced on the programme, and this was maintained at interview	Known smoker to pharmacist – pharmacist asked if she wanted to join the programme
PPT4	Short/Behavioural	M	Quit on the programme and still abstinent at interview	Saw the poster advertising programme to quit and asked to be enrolled
PPT5	Short/Self-help	M	Unsuccessful	Known smoker to pharmacist – pharmacist asked if she wanted to join the programme

These five participants were generally positive about the programme and reported that it had been helpful. Specific aspects of the programme highlighted were the length of time that NRT was available at minimal cost, support from the pharmacist and CO monitoring. They had no changes to suggest to its content or format and reported that they would recommend the programme to others. One exception was a participant in the self-help arm who was not happy to be just given “assignments”. However, he also had no changes to suggest and although he was unsuccessful felt that this was just one of many approaches he had tried that didn’t work.

Generally, there was little evidence that the participants viewed the reduction programme as being fundamentally different to standard stop smoking programmes. The programme was seen as another opportunity to try and quit, and participants did not hold strong opinions that being able to gradually reduce was crucial to success. Rather than strictly following the schedule of the programme, participants who had successfully quit reported that they used the NRT to either quit abruptly or rapidly reduce to quitting. Others had tried to follow the reduction schedules, but there was also a suggestion this hadn’t been done strictly.

### Quantitative efficacy measures

#### Smoking cessation outcomes

Ten participants (one behavioural/standard, three self-help/standard, two behavioural/short, four self-help/short) transferred onto a smoking cessation programme, and set a quit date. Six of these sustained abstinence for four weeks but all had relapsed by six months after their quit date (Tables 9 and 10). One of the six 4 week abstainers was biochemically verified by exhaled CO. There was no difference in the rate of self-report four-week abstinence in the short programme compared with standard length (RR 95%CI 1.00 (0.24, 4.10)) and a non-significant 56% reduction in floating four-week abstinence with behavioural support compared with self-help (RR 95%CI 0.44 (0.10, 1.95)). The corresponding odds ratios (95%CIs) from the generalised linear mixed model were 1.05 (0.20, 6.00) and 0.48 (0.09, 2.69).

**Table 9:** Reduction and cessation outcomes for short length programme compared to standard length programme

Outcome	Short length programme (*n* = 34)	Standard length programme (*n* = 34)		
			RR (95%CI)	RD (95% CI)
Any sustained reduction (n)	4	2	2.00 (0.46, 8.95)	0.06 (−0.09, 0.22)
>50% sustained reduction (n)	3	1	3.00 (0.45, 20.44)	0.06 (−0.07, 0.20)
Floating 4 week cessation (n)	3	3	1.00 (0.24, 4.10)	0.00 (−0.16, 0.16)
Floating 6 months cessation (n)	0	0	N/A	N/A
			Mean difference (SD)	P
Mean (SD) change in cpd baseline – 12 months (baseline carried forward)	−2.29 (7.9)	−2.26 (5.4)	−0.03 (1.65)	0.98
Mean (SD) change in cpd baseline – 12 months (last observation carried forward)	−6.24 (8.81)	−8.41 (9.22)	2.17 (2.19)	0.32
Median (range) change in cpd baseline – 12 months (baseline carried forward)	0 (−30, 20)	0 (−20, 0)		
Median (range) change in cpd baseline – 12 months (last observation carried forward)	−5 (−30, 20)	−8 (−35, 18)

**Table 10:** Reduction and cessation outcomes for behavioural support compared to self-help

Outcome	Behavioural support (*n* = 36)	Self - help (*n* = 32)		
			RR (95% CI)	RD (95% CI)
Any sustained reduction (n)	4	2	1.78 (0.41, 7.97)	0.05 (−0.11, 0.2)
>50% sustained reduction (n)	3	1	2.67 (0.4, 18.19)	0.05 (−0.09, 0.19)
Floating 4 week cessation (n)	2	4	0.44 (0.10, 1.95)	−0.07 (−0.24, 0.08)
Floating 6 months cessation (n)	0	0	N/A	N/A
			Mean difference (SD)	P
Mean (SD) change in cpd baseline – 12 months (baseline carried forward)	−1.58 (7.2)	−3.36 (6.14)	1.48 (1.64)	0.37
Mean (SD) change in cpd baseline – 12 months (last observation carried forward)	−7.31 (8.98)	−7.34 (9.21)	0.04 (2.21)	0.98
Median (range) change in cpd baseline – 12 months (baseline carried forward)	0 (−30, 20)	0 (−21, 0)		
Median (range) change in cpd baseline – 12 months (last observation carried forward)	−5.5 (−30, 20)	−7 (−35, 18)		

#### Smoking reduction outcomes

Six participants smoked fewer cigarettes between months 9–12 than at baseline, four of whom reduced to less than 50% of baseline consumption. There was a non-significant two-fold increase in any sustained reduction in participants in the short length programme compared with standard length (RR 95%CI 2.00 (0.46, 8.92) or with the random effects model OR 2.51 (0.33, 19.11)) and a non-significant 78% increase with behavioural support compared with self-help (RR 95%CI 1.78 (0.41, 7.97) or with the random effects model OR 3.05 (0.38, 24.76)). Similarly, there was a non-significant increase in >50% sustained abstinence in the short programme compared with standard length, and a non-significant increase with behavioural support compared with self-help ((RR 95% CI 3.00 (0.45, 20.44) and OR 3.05 (0.38, 24.76)) and RR 95% CI 2.67 (0.40, 18.19) and OR 2.51 (0.33, 19.11) respectively).

## Discussion

There was strong evidence that a future trial of this kind of intervention would be unfeasible. It proved hard to recruit as intended, using naturally occurring conversations in a pharmacy, and asking doctors to write to patients that smoked and offer the pharmacy service did little to change this. Even though pharmacists felt the programme could potentially support smokers who find it difficult to quit, they became largely disengaged with the study, partly for reasons related to the trial documentation and partly that they were not comfortable with randomising participants. They also saw abrupt cessation as a better goal and one they were more comfortable supporting and were very motivated to provide smoking cessation and reduction support to their clients. Participants too seemed not to welcome the opportunity to reduce as such, rather they saw it as a means of achieving abstinence or preferred a very short reduction phase. Most participants dropped out of treatment after the first month or two with only a few following the treatment programme through the full nine months. Follow-up of participants as part of the trial procedure using the internet was almost never successful and follow-up by telephone became unsuccessful as participants dropped out of the programme. There was no evidence that either more rapid reduction or behavioural support improved the rate of abstinence or of reduction, but the effect estimates were imprecise.

There were several important strengths of the trial. The first is that we developed intervention programmes based on existing evidence of effectiveness of smoking reduction programmes. The evidence on NRT was clear [4, 5], but the evidence that behavioural support was effective is less clear and the evidence that shorter programmes are more effective is similarly uncertain [7]. That said, it appears that following a structured programme enhances the reduction and cessation so we developed easy to use methods and tools to support people doing this at home. We developed a well-received training programme for training pharmacists and judged that all of them were competent after completing it. We supported pharmacists in delivering this new programme and in the processes of the trial. We also developed a comprehensive plan to evaluate several aspects of implementing this trial [13] and produced clear-cut results as a consequence.

There were some limitations of the trial. Key staff at our research centre left during the course of the trial, which meant that our support programme was perhaps not as rigorous as it might have been. In addition, there were technical problems with the trial database, which meant that follow-up was not as rigorous as it might have been. We completed fewer interviews than anticipated and it could well be that, in particular, we did not obtain a comprehensive picture of the range of views. That said, it is clear to us that the lack of enthusiasm of potential participants and pharmacists was as a result of trying to implement the programmes as intended, rather than difficulties arising from the support provided by the trial team. The trial itself was potentially underpowered to detect effects on abstinence and reduction of the size we might have anticipated, though this was always a secondary concern compared with the aim of assessing feasibility. However, having failed to recruit the requisite number of participants, the trial provides little direct evidence on the relative effectiveness of behavioural support or speed of reduction in smoking reduction programmes.

These results have implications for public health research and practice. This trial took place in inner-city Birmingham, one of the most socioeconomically deprived areas of England. It appears that, at least in the context of a socioeconomically deprived inner-city, smoking reduction programmes supported by nicotine replacement are not popular with potential users. Feedback from both pharmacists and smokers suggested that they preferred very rapid reduction programmes focused on a short-term reduction goal with cessation clearly intended. Since this trial started, smoking reduction using electronic cigarettes has become very popular [17], though largely unsupported by health professionals such as pharmacists, as we tried here. The results may have implications for trials with pharmacists. It was clear here that some were unprepared to randomise participants or ignored the randomisation and provided what they thought was best interest of their patients. Many others expressed concern about randomisation to self-help. We had tried to counter this by providing training about the basis for randomisation and why it was important for proving what the pharmacists might believe to be true, but it was clear this training failed in some cases. Future trials in any context may need to pay particular attention to assessing equipoise and further training on this point as well as closer supervision, which, this trial shows, cannot be taken for granted.

Although this trial met with difficulties in recruiting, randomising, and delivering care, other trials based in pharmacies have had much greater success in these areas. A systematic review of pharmacy delivered public health programmes testing the effectiveness of interventions on smoking, excess alcohol consumption, and weight loss programmes found 19 studies including 12 randomised trials [18]. Generally, these programmes ran well without problems enacting randomisation and recruited to target. There was clear evidence that, compared with no intervention, pharmacy-delivered smoking cessation support was effective but no evidence it was more effective than other active cessation support interventions. The poor enactment of this trial may reflect both pharmacists’ and members of the public’s lukewarm reaction to smoking reduction. A series of pilots of new models of smoking cessation support that offered smoking reduction programmes in the NHS resulted in low uptake, with nearly all service users opting for abrupt cessation, even among groups such as people on methadone maintenance programmes [19]. Thus, it appears that part of the failure of this programme related to the aim of delivering a smoking reduction programme, which few people wanted.

## Conclusion

Pharmacists were willing to support patients to reduce their smoking in theory, but a well-designed training programme failed to motivate pharmacists to deliver the programme as intended. Opportunistic identification of potential participants by pharmacists, with additional advertisement to smokers by GPs did not result in attainment of the recruitment target. This programme as implemented is not feasible in routine practice at pharmacies and suggests that short and simple programmes would be more readily engaged with in this context.
