# Erratum to: A mixed methods feasibility study of nicotine-assisted smoking reduction programmes delivered by community pharmacists – The RedPharm study

**DOI:** 10.1186/s12889-017-4204-0

**Published:** 2017-03-31

**Authors:** Amanda Farley, Sarah Tearne, Taina Taskila, Rachel H. Williams, Susan MacAskill, Jean-Francois Etter, Paul Aveyard

**Affiliations:** 1 Institute of Applied Health Sciences, University of Birmingham, Edgbaston, B15 2TT, Birmingham, UK; 2 Nuffield Department of Primary Care Health Sciences, University of Oxford, Radcliffe Primary Care Building, Radcliffe Observatory Quarter, Woodstock Road, Oxford, OX2 6GG UK; 3 Department of Family Care & Mental Health, Faculty of Education & Health, University of Greenwich, London, UK; 4 Institute for Social Marketing, University of Stirling, Scotland, FK9 4LA UK; 5 Faculty of Medicine, University of Geneva, Geneva, Switzerland

## Erratum

Following publication of this article [1], it has come to our attention that the author, Susan MacAskill, has had her name captured incorrectly. The correct spelling is the aforementioned.
